# Understanding Long COVID; Mitochondrial Health and Adaptation—Old Pathways, New Problems

**DOI:** 10.3390/biomedicines10123113

**Published:** 2022-12-02

**Authors:** Alistair V. W. Nunn, Geoffrey W. Guy, Wolfgang Brysch, Jimmy D. Bell

**Affiliations:** 1 Research Centre for Optimal Health, Department of Life Sciences, University of Westminster, London W1W 6UW, UK; 2 The Guy Foundation, Chedington Court, Beaminster, Dorset DT8 3HY, UK; 3 MetrioPharm AG, Europaallee 41, 8004 Zurich, Switzerland

**Keywords:** SARS-CoV-2, mitochondria, Kreb’s cycle, metabolic flexibility, hormesis, inflammation, long COVID, lifestyle, platelets

## Abstract

Many people infected with the SARS-CoV-2 suffer long-term symptoms, such as “brain fog”, fatigue and clotting problems. Explanations for “long COVID” include immune imbalance, incomplete viral clearance and potentially, mitochondrial dysfunction. As conditions with sub-optimal mitochondrial function are associated with initial severity of the disease, their prior health could be key in resistance to long COVID and recovery. The SARs virus redirects host metabolism towards replication; in response, the host can metabolically react to control the virus. Resolution is normally achieved after viral clearance as the initial stress activates a hormetic negative feedback mechanism. It is therefore possible that, in some individuals with prior sub-optimal mitochondrial function, the virus can “tip” the host into a chronic inflammatory cycle. This might explain the main symptoms, including platelet dysfunction. Long COVID could thus be described as a virally induced chronic and self-perpetuating metabolically imbalanced non-resolving state characterised by mitochondrial dysfunction, where reactive oxygen species continually drive inflammation and a shift towards glycolysis. This would suggest that a sufferer’s metabolism needs to be “tipped” back using a stimulus, such as physical activity, calorie restriction, or chemical compounds that mimic these by enhancing mitochondrial function, perhaps in combination with inhibitors that quell the inflammatory response.

## 1. Introduction

Long COVID, or post-acute sequelae of COVID-19 (PASC), could be affecting more than two million people in the UK [1]. Globally up to 43% of people who had proven infection display symptoms for 2–3 months or more [2]. However, due to “slippery” definitions others have suggested the effectiveness of preventing long COVID with vaccination could vary from 15 to 50%, while the overall prevalence could be anywhere between 5 and 50% [3]. What is clear is that however it is defined, severity of infection does seem to be associated with an increased risk of long COVID, and although prior vaccination can certainly reduce this risk, it does not completely in those who develop a break through infection [4]. The most common symptom seems to be fatigue, closely followed by depression, anxiety and cognitive dysfunction, sleep disorders, breathlessness, and smell/taste loss [5]. This is also linked to decreased exercise capacity, which is only partly explained by the expected deconditioning that occurs when someone has been ill [6], additionally, there is also evidence of persistent clotting problems [7]. It is therefore not surprising that long COVID is clearly having a big impact on the quality of life for those that suffer from it [8].

A recent meta-analysis of 2020–2021 data suggests that globally, more than 14 million people experienced one or more of the key symptoms three months post-infection, with most cases arising from people who had milder infections. At 12 months, 15.1% still had not recovered [9]. Children can also develop it, and worryingly, even in those who have had mild or no symptoms [10]. Thus, long COVID could be becoming a global emergency [11]—especially if many years later, it has accelerated the ageing process, leading to rising incidence of cancer, metabolic syndrome and Alzheimer’s. Certainly there is evidence for long-term neurological complications after one year [12], which agrees with data that the virus can infect brain cells [13]. Given the already huge cost burden associated with obesity and dementia, this may be something that needs to be addressed.

There is thus a pressing need to truly understand the pathophysiology as it might provide clues to treatment and prevention. To date, the candidate mechanisms include persistence of the virus, reactivation of other viruses, induced autoimmunity, tissue damage inducing persistent inflammation and formation of microthrombi [5]. With regards the latter hypothesis, COVID-19 mortality is strongly related to coagulopathy and disseminated intravascular coagulation (DIC) and cytokine storms, which are associated with thrombosis and bleeding, as well as thrombocytopenia [14]. Critically, this could also play a role in long COVID as even in those who have had mild symptoms, clotting problems still occur and appear to be associated with increased circulating fibrinolytic resistance amyloid complexes, in particular, the acute phase protein, serum amyloid A (SAA4) and α(2)-antiplasmin (α2AP) [7].

There is thus a potentially informative overlap with the long-researched condition, myalgic encephalomyelitis/chronic fatigue syndrome (ME/CFS), which is also associated with microclots [15]. CFS often occurs after viral infections and it has been proposed that some of the same mechanisms, for instance, involving neuroinflammation, could have common origins [16]. Furthermore, the severity of the initial disease is strongly associated with metabolic perturbation, for instance in the Kreb’s cycle and central carbon metabolism, which is associated with dysregulation and imbalance of the various cells of the immune system [17]. Tellingly, CFS is potentially also associated with decreased mitochondrial function [18]. As mitochondria are critical to platelet homeostasis [19], the idea that preserving platelet mitochondrial function could be important in COVID-19 pathogenesis has also been raised in the context of microbiota dysfunction and extracellular mitochondria swapping [20].

Interestingly, there is emerging evidence that many, maybe all cells, including platelets, can either donate or receive mitochondria and that the mitochondria themselves act as signals, for instance when damaged and inflammatory, or healthy, and potentially anti-inflammatory [21]. Thus, as prior systemic mitochondrial health could be a strong factor dictating the severity of the acute disease someone develops [22,23,24,25], it could also be indicative of whether someone is more liable to develop long COVID.

Certainly, data are now indicating that the plasma proteomic signature in the acute setting can predict who is more likely to develop long-term symptoms, even in those not hospitalised. For instance, one of the most predictive components are raised levels of the iron-sulphur cluster biogenesis protein (HSCB), which could hint at increased iron levels and observed association with hyperferritinaemic state and tissue damage, suggesting possible iron metabolism dysfunction [26]. Indeed, hepatic iron deposition in COVID-19 patients has been reported, with many of the increased number of ferritin particles being associated with mitochondria [27]. As mitochondria are key in the synthesis of iron sulphur proteins [28], and deficiencies in mitochondrial complex 1 can lead to iron metabolism dysfunction [29], this might suggest compromised mitochondrial function could also help explain these observations.

It is thus possible that the virus in some people “tips” their metabolism into a new state, which is characterised by an inability to restore optimal mitochondrial function and remains biased towards glycolysis. The reasons for this are likely many, but as indicated, poor prior mitochondrial health and robustness are important. This could be the result of prior low aerobic fitness, underlying inflammation related to the metabolic syndrome, as well as other viral infections, co-morbidities or genetic defects that give rise to oxidative stress. As to an explanation that might underpin this tipping point, the concept of mitochondrial DNA editing during oxidative stress to decrease the electron transport chain (ETC) components and thus reactive oxygen species (ROS) [30], when combined with the reasons why mitochondria retain their own genes [31] might provide a hint due to the loss of mtDNA copy number as a marker for a lower mitochondrial reserve and loss of metabolic flexibility.

Put simply, it could be possible that if somebody’s mitochondrial reserve is not great enough, and the system gets overloaded, it tries to reduce oxidative stress by decreasing mitochondrial function still further. For example, a reduced mtDNA copy number is associated with increased incidence of type 2 diabetes and the metabolic syndrome and thus, insulin resistance [32]. Certainly, the severity of the initial SARS-CoV-2 infection is positively associated with greater circulating mtDNA, as this is a key marker of inflammatory processes [33]. Indeed, it has been suggested that excessive release of mtDNA could be a key pathogenic mechanism with this virus [34], which could be related to the observation that cells can expel damaged mitochondria via the process of mitocytosis [35]. In this light, it seems that corona viruses, including SARS-CoV-2, can directly inhibit and modulate mitochondrial function, inhibiting energy production and inducing apoptosis; it has been suggested that COVID-19 pneumonia is triggered by a SARS-CoV-2 mitochondriopathy [36]. This suggests that if someone’s initial mitochondrial reserve is compromised, then the virus could make this worse.

In this regard, it could be informative that a fundamental piece of metabolism, the mitochondrial Kreb’s cycle, which may have arisen in an alkaline thermal vent at the very beginnings of life, highlights the importance of balancing energy production and biosynthesis with the availability of oxygen. A key component of the evolution of complex life is that different cells, and perhaps even different mitochondria within the same cell, can have the Kreb’s cycle operating in a variety of different modes, which for the whole, are synergistic [37]. The similarities between inflammation and growth are perhaps informative here: there is much to be learnt from both the Warburg [38] and reverse Warburg effects, which provides insight into both cancer and Alzheimer’s [39]. Indeed, components of the Kreb’s cycle are not just central to energy and biosynthetic metabolism, but also signalling, inflammation and immunity [40]. This could be especially important as these metabolites, for instance, via epigenetics, could modulate the ageing process [41]. Critically, this virus may also modulate epigenetics, as one of its proteins, encoded by ORF8, could be a histone mimic [42]. This further suggests that SARS-CoV-2 is modulating, at a very basic level, the core metabolism of its host.

However, interpreting what these changes in metabolism mean is difficult, as host cells also modulate metabolism to fight pathogens. For example, altering lipids to prevent pathogen entry, manipulating iron metabolism, and modulating mitochondria to starve them of metabolites. What this suggests is the metabolic perturbations seen in infections may not just be the direct effect of the pathogen, but the system’s response to it [43]. Certainly, cells rapidly alter mitochondrial structure in response to viruses, while viruses themselves can also modulate mitochondrial dynamics [44]. Furthermore, for most infected people, the immune system is not just fighting off COVID-19, but other pathogens too, as well as managing its relationship with its more friendly microbiota. Mitochondria are also key in modulating circadian rhythms, whose disruption is linked to disease [45]. In short, in the evolutionary arms race between viruses and their hosts, not only have mitochondria become a target, but they are also “weaponised” by the host.

In this paper, we discuss mitochondrial function and the evolutionary origins of metabolism and the Kreb’s cycle and interlace it with the emerging literature that either directly, or indirectly, SARS-CoV-2 is manipulating metabolism and suggest the possibility that “long COVID” is a syndrome, in part, related to an overloaded mitochondrial compartment resulting in metabolic inflexibility and an inability to restore homeostasis. The predisposing sub-optimal mitochondrial phenotype could be the result of either a comorbidity, age and/or a prior poor lifestyle resulting in a reduced mitochondrial reserve and fitness. Worryingly, this phenotype could potentially be associated with an accelerated ageing process. Indeed, COVID-19 has already worsened period life expectancy, a measure of population health—especially in countries already exhibiting a pre-existing mortality crisis, such as the United States [46]. Given that vaccination seems to reduce visits to the doctor with symptoms of long COVID [47], this further supports not just vaccination programs, but also enhancing fitness and a reduction in obesity rates to prevent a possible future crisis. Although we will not discuss genetics per se, the emerging link between APOE genotype and the severity of COVID-19 could also be informative. For instance, the APOE2 and APOE4 alleles are associated with alterations in lipid metabolism and a worse outcome [48]. The link here is that APOE4 is associated with reduced mitochondrial function [49]. An important implication of the thesis in this paper is that enhancing mitochondrial health could be key in both prevention and treatment of long COVID. Certainly, the concept of an obesity/viral “syndemic” is becoming established, as there seems to be a clear link with COVID-19 deaths and an unhealthy lifestyle [50]. We also apply the mitochondrial concept to the platelet, which might also begin to explain the association with clotting problems.

## 2. From Alkaline Vents to Mitochondria and SARS-CoV-2

A key principle of extant, or modern-day biochemistry, is that its roots can be traced nearly all the way back to the beginnings of life. Evidence is that life evolved based on the earth’s early geochemistry [51], and can be viewed as the descent of the electron [52]. As to where, one of the strongest theories, due to the existence of the proton gradient and universality of both the electron transport chain (ETC), and the existence of the Kreb’s cycle, is the deep-sea alkaline thermal vent. One of the fundamental theories that falls out of this is that originally, something like the Kreb’s cycle started as a biosynthetic system, utilising hydrogen and carbon dioxide to create complex chemistry. At some point during evolution, this system then evolved to work in reverse to extract energy from complex molecules and generate a proton gradient. In short, the original “forward” mode of the Kreb’s cycle was biosynthetic, not energy producing. Today, of course, it is viewed the other way around [37].

This simple concept can have a profound impact on how we view what viruses, such as COVID-19, might do to our metabolism and what our metabolism, in trying to defend against the virus, might also do. For instance, at a very basic level, the virus needs to reprogramme metabolism back to a biosynthetic mode, which could be a very ancient strategy indeed, while manipulating the more modern immune system. If the original direction of the Kreb’s cycle was biosynthetic, in effect, using the energy in a proton gradient to build molecules, then a later evolutionary step was that it could go into reverse by taking energy out of molecules to generate a gradient. A mechanism for doing this relies on electrons flowing down to a highly electro-negative compound or element, such as oxygen. This of course is reflected in modern day metabolism in the relationship between inflammation, biosynthesis, and hypoxia, and thus, glycolysis and the mitochondrion.

### 2.1. Beyond the Powerhouse—Mitochondria Do a Lot More Than Produce Energy

**Figure 1:**
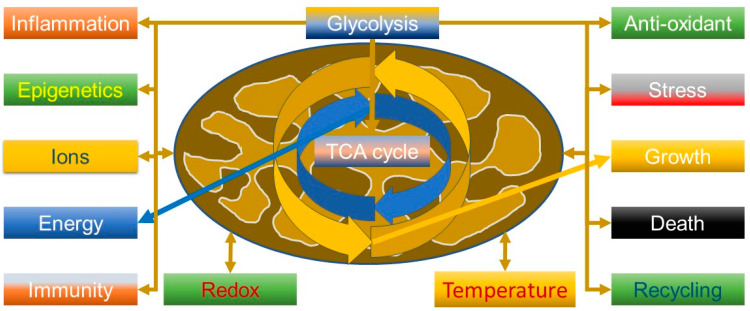
Some of the many roles of the mitochondrion and the centrality of glycolysis and the tricarboxylic acid cycle (TCA, also known as the Kreb’s cycle).

An important aspect to understanding the virus is that mitochondria are not just simply “powerhouses” of the cell but have many more functions (Figure 1), including calcium and ROS homeostasis, which could be predicted from when they got together with an Archean to become a eukaryote [53]. The Kreb’s cycle thus has many functions. For example, succinate is involved in signalling, tumorigenesis, inflammation, redox and epigenetics [54], and can suppress influenza viral replication by succinlyation of viral nucleoproteins in the nucleus [55]. It is thus relevant that tumour necrosis factor (TNF) can increase succinate production, driving reverse electron transport (RET) through complex 1 and generating ROS; this can be a powerful anti-pathogen mechanism, but also pathological if not controlled and can be modulated by metformin, a known complex 1 inhibitor [56].

Succinate builds up during hypoxia and is important in generating ROS, for instance, during heavy exercise—and is thus a key signalling moiety when phosphorylation cannot occur. ROS production is thus closely related to mitochondrial membrane potential. Critically, much of the ROS generated is converted into hydrogen peroxide, which is a signalling molecule and readily diffusible. However, less appreciated is that mitochondria also contain several powerful ROS reduction systems (RDS), some of which rely heavily on NADPH, indicating they can act as net ROS sinks. For example, they can effectively remove external hydrogen peroxide, especially a higher concentrations; they are therefore central to ROS-based signalling as they can act as both a ROS sink and a producer [57]. Critically, it seems mitochondria from different tissues have different ways, and abilities, of acting as sinks of ROS, suggesting cell-specific functions [58,59].

Overall, this means that mitochondria tightly control ROS, but the extent may vary from cell to cell. It is thus possible that the antioxidant systems are not simply there to offset uncontrolled ROS production, but are a fundamental part of signalling, and are closely linked to bioenergetic status [60]. Tellingly, it now seems that these systems could be important in determining the longevity of a species, in effect, the ability of an organism’s mitochondria to consume ROS determines how long it lives [61]. A key way to view this is that the redox couples in the mitochondrion are closely linked to substrate oxidation, and thus to the anti-oxidant system (e.g., generation of NADPH), which has led to the redox-optimised ROS balance hypothesis and the idea that mitochondria evolved to operate at an intermediate redox state, where energy production is maximised, but ROS production minimised—if the system is too oxidised, or reduced, ROS increases [62]. In this regard, the ability of bats to tolerate viruses, including SARs, is perhaps relevant, as they are extremely long lived, yet very active; one aspect of their metabolism is the ability to manage oxidative stress and inflammation—this could be related to their flight capacity and mitochondrial function [63].

This does indicate that not only is mitochondrial health an important determinator of resistance to this virus, but that resistance to oxidative stress might vary from cell to cell, which may determine the outcome depending on which organs and cells the virus has affected, either directly, or indirectly. It is well described that modulation of Kreb’s cycle intermediates by viruses is key in how they replicate, as this supplies the metabolites they need, such as citric acid, but it also controls the immune system. For instance, fumarate and itaconate have anti-viral activity and are immunomodulators and can control the nuclear factor erythroid 2–related factor 2 (Nrf2), a key regulator of resistance to oxidative stress, while the latter can inhibit succinate dehydrogenase (SDH). In contrast, as discussed, succinate is viewed as being pro-inflammatory [64]. Interestingly, activators of Nrf2, such as dimethyl fumarate have indicated that SARS-CoV-2 suppresses the Nrf2 pathway, and so indicate possible therapeutic approaches—as this pathway is not only anti-inflammatory but appears to have a distinct anti-viral function [65].

Certainly, one metabolomic study of COVID-19 patients indicated that they had higher succinate and lactic acid, but lower citric acid levels compared to healthy controls [66]—which is strongly suggestive of Kreb’s cycle modulation. Other metabolomic studies have also shown changes in the Kreb’s cycle indicating mitochondrial dysfunction, with increases in succinate [67,68]. One study did not show any change, but could have been explained as the patients were receiving intense respiratory therapy, suggesting hypoxia may be important [69]. Considering this latter study, another showed that in more severe cases there was an indication of modified amino acid metabolism that was commensurate with hypoxia and a shift in mitochondrial metabolism [70]. It may therefore be relevant that platelets express the succinate receptor, SUCNR1, which can stimulate platelet activation [71]. We will discuss platelet metabolism in more detail later in the paper.

### 2.2. SARS-CoV-2 Can Modulate Mitochondria and Glycolysis

Data suggest that a SARS-CoV-2 membrane protein can directly cause mitochondrial apoptosis, which may lead to enhanced lung injury [72]. In general, coronaviruses including SARS-CoV-2 have been found to modulate mitochondria. In several models involving lung cells, the virus was found to inhibit the formation of components of the ETC, in particular complex 1, and induce mitochondrial permeability transition (MTP), reduce mitochondrial membrane potential, disrupt ATP synthase function, enhance mitochondrial fission, and induce apoptosis. In the lungs, this could inhibit the hypoxic pulmonary constriction response (HPV) by inhibiting oxygen sensing. In short, in severe COVID-19, the hypoxemia associated with lung injury could be a viral mitochondriopathy [36].

It has also been found that in the leukocytes of patients with post COVID-19 sequalae there is evidence of decreased mitochondrial membrane potential [73], while in an elderly population, a decreased mitochondrial membrane potential seemed to associate with an increased susceptibility and vulnerability to the virus [74]. Another observation is that in placental samples from infected patients, SARS-CoV-2 RNA co-localised with mitochondria, which was associated with altered mitochondrial networks [75] and although one study did not find any changes in mitochondrial long non-coding RNAs during infection, during recovery, there was a persistence change in small mitochondrial RNAs [76]. This of course might suggest that there might be host mitochondrial transcriptome responses to SARS-CoV-2, as there are in response to other viruses. In one study, the virus did not appear to significantly alter mtDNA gene or mitochondrial antiviral signalling protein (MAVs) expression but did seem to downregulate nuclear encoded mitochondrial genes [77].

The role of SARS-CoV-1 protein ORF3b is also suggestive, as it has been found to target mitochondria and modulate interferon production [78], while ORF3a from SARS-CoV-2, via its effects on mitochondria, seems to play a role in activation of the hypoxia-inducible factor-1α (HIF-1α) that enhances viral infectivity [79]. Interestingly, researchers using a fruit fly model found that the viral Nsp6 protein can damage hearts, which is of great clinical interest because of the association of COVID-19 with cardiovascular problems. Their key finding was that it induced a switch to glycolysis, which was associated with mitochondrial damage, and activation of the Myc pathway. Critically, inhibitors of glycolysis could reduce the severity of the damage both to *Drosophila* hearts and mouse cardiomyocytes, suggesting a potential clinical strategy [80].

Considering the ability of the virus to induce metabolic reprogramming of its host, a further link is also suggested by the emerging pleiotropic roles of vacuolar-ATPase (V-ATPase) and viruses. V-ATPase is a large proton pumping turbine consisting of many different, and interchangeable subunits that vary according to its place in and the function of a particular cell. Its most readily identifiable function is to use ATP to acidify compartments, but it has many more roles. For example, its association with the mammalian target of rapamycin (mTOR), the AMP-activated protein kinase (AMPK) and transient receptor potential V-type channels (TRPV) indicate a role in nutrient sensing and modulating energy levels. In essence, with increased glucose, it is involved in activating mTOR, but when glucose levels fall, it can inactivate mTOR, activating AMPK and via TRPV, interact with the endoplasmic reticulum. Equally, when amino acid levels rise, it can also activate mTOR, but when they fall, it can inactivate it [81]. In effect, it seems to be part of the system for switching from glycolysis to oxidative phosphorylation when the organism is under starvation and vice versa, thus shifting from anabolism and catabolism and back again. These pathways are intimately coupled to control of mitochondrial function [82]. Furthermore, it also seems to be modulated by oxidative stress via Oxr1, which induces its disassembly [83], while the targeting of the different isoforms is dependent on their phosphatidylinositol lipid composition, which is related to the pH of the organelle [84].

**Figure 2:**
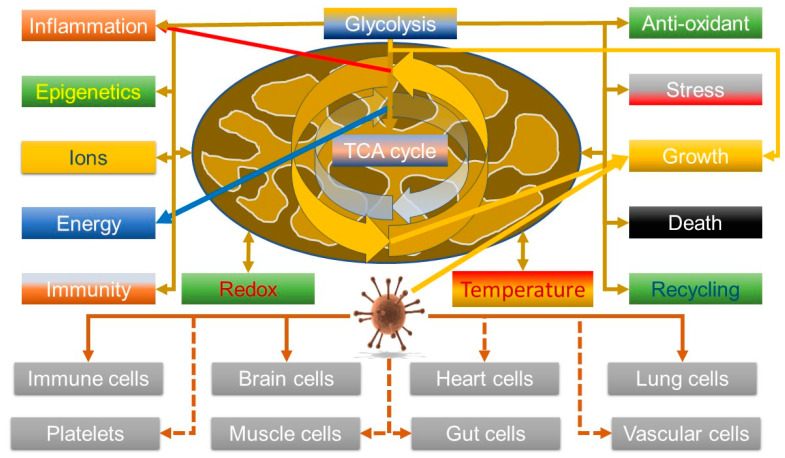
Potential viral manipulation of mitochondrial function in multiple cell types could be allied with symptoms of long COVID; the virus manipulates the system towards growth pathways that are similar to inflammatory pathways, which are also how the host tries to get rid of the virus.

It is thus relevant that data are indicating that the virus can manipulate V-ATPase—increasing its levels [85] and that its entry into cells can be synergistically blocked by inhibiting both V-ATPase, using bafilomycin A1, and the cell surface transmembrane protease serine 2 (TMPRSS2), using Camostat. Hence, viral entry can be achieved by both the plasma membrane route involving TMPRSS2 and an endosomal route, which explains why some inhibitors, such as chloroquine, have not been that effective [86]. So, although this could be explained by a mechanism that simply inhibits endosomal acidification, it might also hint that it could affect metabolic reprogramming. In this light, given that COVID-19 seems to be associated with a hyper-ferritinaemia and in some cases, hepatic failure associated with iron overload [27], it may be relevant that V-ATPase is also key in controlling HIF1α by modulating iron levels; V-ATPase depletion/inhibition enhances HIF1α activity by depleting transferrin uptake [87]. However, the effects V-ATPase on HIF, and thus glycolysis, are nuanced; although its inhibition can activate glycolysis, so enhancing cell survival, it can also kill cancer cells due to proton build up in the cytosol [88]. So, as HIF is known to be important in viral defence [89], this might suggest that this could be a viral defence mechanism. A somewhat sobering thought is that most oncogenic viruses seem to upregulate HIF as well—although the direction of the causality is unclear [90], but it does suggest that SARS-CoV-2 might heighten the risk of cancer. Overall, the emerging data, both about viruses in general, and this one, do seem to support it can modulate mitochondrial function. Figure 2 summarises the link between the virus and mitochondrial function.

### 2.3. Taking the Viral Viewpoint; Is Hypoxia Good or Bad for the Virus?

As indicated, oxygen levels and metabolism are very tightly integrated. Interestingly, many viruses are sensitive to oxygen tension, but the outcome is very context dependent, and the response varies with the species and the host cell: hypoxia enhances EBV but suppresses influenza replication. It has been shown, at least in respiratory tract cells, that activation of the HIF pathway can downregulate the angiotensin converting enzyme (ACE2) and that post-entry steps into a cell are oxygen sensitive for COVID-19—hindering its replication, at least in these cells [91]. However, others have found that HIF can promote COVID-19 infection, worsening inflammation, with a suggested mechanism involving ORF3a directly damaging mitochondria [79]. This is perhaps further reinforced by the finding that elevated glucose levels also favour infection, which is accentuated by a HIF-dependent mechanism in lung monocytes that enhances glycolysis that can inhibit T cell responses [92]. This suggests that while in some cells an anti-viral HIF mechanism is activated, in other cells, it could favour the virus.

Given the immense amount of time that viruses and their hosts have been co-evolving in an almost eternal arms race, it would be no surprise that the virus has evolved to utilise the very metabolic reprogramming that its host uses to try and get rid of it. This would suggest that trying to untangle what the host’s metabolic defence program is and what is the viruses’ manipulation of it is difficult. For example, a Warburg shift could benefit both the host and the virus, depending on context, but it does raise the possibility that induction of mild hypoxia might also favour the virus, and has thus been adopted by the virus through evolution. However, in terms of the Kreb’s cycle, this does suggest a bias towards a biosynthetic mode that could aid the virus. Whether this effect is enhanced in the presence of pre-existing sub-optimal mitochondrial function is thus perhaps an interesting question, as cellular metabolism might be slightly more reliant on glycolysis in these circumstances. The clear thrombotic state seen in many patients could be an example of a positive reinforcement feedback loop that perpetuates a very damaging inflammatory cycle, hinting, potentially at a tipping point. This would especially be the case if the virus damages mitochondria.

### 2.4. Viruses, Mitochondria and Lipids; a Nutritional Arms Race

The previous discussion touches upon a possible problem in dissecting out what is a direct viral effect, and potentially, a host reaction. It is thus perhaps worth remembering that the host versus virus is a very old arm’s race, and both sides modulate metabolism, especially the Kreb’s cycle and mitochondrial function; this has been called “nutritional immunity” from the host’s perspective. One mechanism is not only to try and starve pathogens of lipids, but for the host to alter membrane structure to make entry more difficult, for instance, by modulating cholesterol and other lipids, such as sphingolipids [43].

One of the functions of a more biosynthetic Kreb’s cycle mode is to produce lipids. It certainly seems that viruses have evolved to usurp this mechanism. It now appears that SARS-CoV-2 is no different as it comprehensively rewires its host lipid metabolism, with a key feature being the formation of lipid droplets. This is supported by clinical observations at the systemic level, which include changes in the apolipoprotein system. Critically, viral replication could be inhibited by small molecule glycerolipid biosynthesis inhibitors [93]. Interestingly, as mentioned in the introduction, the APOE4 allele seems to be associated with impaired neuron-astrocyte coupling of fatty acid metabolism, which is associated with mitochondrial dysfunction and an inability to form lipid droplets and a shift towards glycolysis [94]. This would suggest that differences in lipid metabolism genotype could affect outcome.

So could the virus, by modulating mitochondrial function, affect lipid metabolism? Mitochondria play a key role in lipid metabolism, for instance, via Kreb’s cycle intermediates and NADPH, or by burning fatty acids in respiration, but perhaps less appreciated is that their function is very dependent on their own lipid system, which seems to explain why they contain their own fatty acid synthase (mtFAS), which utilises acetyl CoA to build C8 fatty acids. In fact, it seems that mitochondrial fatty acid synthesis coordinates oxidative metabolism, and its loss leads to severe problems with the production of ETC complexes [95,96], while defects in mitochondrial fatty synthesis are associated with neurodegeneration [97]. Furthermore, NAD kinase (NADK) supports lipogenesis by maintaining a pool of NADPH, which in concert with mtFAS, is key in maintaining mitochondrial mass by controlling acetyl-CoA and peroxisome proliferator-activated receptor gamma one alpha (PGC-1α). As a central component of cellular lipid storage is the lipid droplet, defective mitochondria can lead to lipid accumulation—although this is balanced by their role in both providing energy and precursors for cytosolic fatty acid synthesis [98]. It may also be relevant that isocitrate dehydrogenase 2 (IDH2) deficiency, a key mitochondrial enzyme, seems to be critical in myogenesis and fatty acid metabolism [99].

Putting this together, this virus could induce a shift in mitochondrial function that is linked to altered lipid metabolism. Whether this is direct, or indirect, is not clear; it could again be a phenotype generated by host adaptation to get rid of the virus. It has long been known that one of the trades offs of defence against pathogens is “friendly fire” damage. A classic example of this is perhaps the dyslipidemia and insulin resistance associated with activation of the acute phase response, and thus inflammation, with conditions like atherosclerosis and the metabolic syndrome and lifestyle-induced disease, and the role of peroxisomal proliferating-activated receptors (PPARs) [100].

In this light, there is, perhaps, another relevant observation, and that is that in the kidneys of diabetic mice, peroxisomal succinate production is increased leading to increased lipid accumulation and oxidative stress via suppression of mitochondrial fatty acid oxidation [101]. It seems that succinate is a suppressor the antiviral immune response by modulating the MAVs complex [102].

In summary, there is an evolutionary rationale as to why SARS-CoV-2 would modulate Kreb’s cycle intermediates and lipid metabolism, equally there is also a good reason why the host may do it to get rid of the virus. However, this could be truly ancient indeed, as theories on the earliest proto-metabolism that gave rise to life, certainly indicate that it can lead to the formation of lipids [103].

## 3. Long COVID and Mitochondria—A Tipping Point

The above discussions do seem to support the idea the both the virus, and the host, will manipulate mitochondrial function; the former to help it replicate, the latter to get rid of the virus. Is there a point where if both operate in the same direction, and mitochondrial function is sub-optimal, the system ends up in a vicious spiral?

Current thinking suggests that long COVID pathology is associated with five key perturbations: persistence of the virus; reactivation of other viruses; induced autoimmunity; tissue damage inducing persistent inflammation; and formation of microthrombi [5]. Certainly, all of these could be involved. However, what is clear is that the metabolic phenotype remains in a sub-optimal homeostatic state, which is commensurate with low grade inflammation. Whether or not this is caused by persistent virus, or other viruses, or even bits of virus, or simply that the host is “stuck” in a kind of “defence” mode, and cannot for some reason resolve itself, is unclear. Whatever is happening, it appears to be associated with a reprogramming of mitochondrial function, which is likely linked, because of the importance of mitochondria in epigenetics, to a kind of epigenetic stress state. As to why the system in some people “tips” into this state could be due to a prior and already altered mitochondrial status, perhaps coupled to chronic inflammation induced by a poor lifestyle, age and/or another co-morbidity.

In this section, we review the evidence linking mitochondrial function and the longer-term effects of COVID-19 and highlight some less obvious links. One possibility is that through evolution, the virus has evolved to utilise the very metabolic phenotype that the host normally uses to defend against the virus. For example, it thrives, in some tissues, due to a mildly hypoxic inflammatory environment where the Kreb’s cycle is more likely to be in biosynthetic mode, which is very similar to what happens in some cells during inflammation.

**Figure 3:**
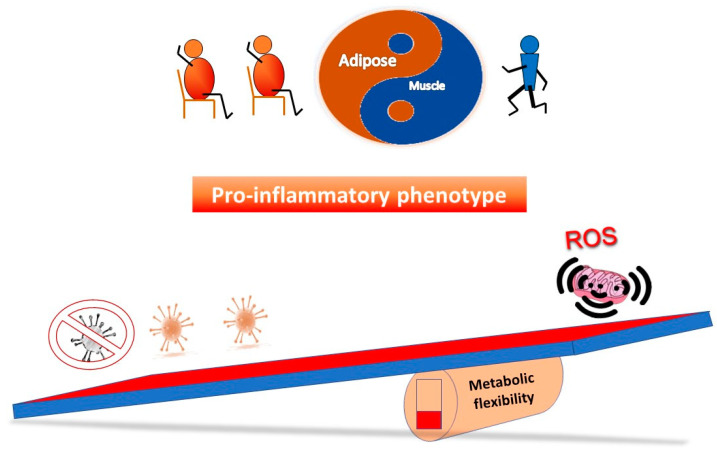
Why a poor lifestyle might lead to a greater likelihood of long COVID. Poor metabolic flexibility, as a result of a lifestyle that does not stimulate optimal mitochondrial function, can result in low grade inflammation that may well further stress mitochondrial function. On exposure to the virus, the system is further tipped towards an inflammatory phenotype, which coupled with sub-optimal mitochondrial function in the immune system, may take longer to either clear the virus and/or resolve the proinflammatory state.

**Figure 4:**
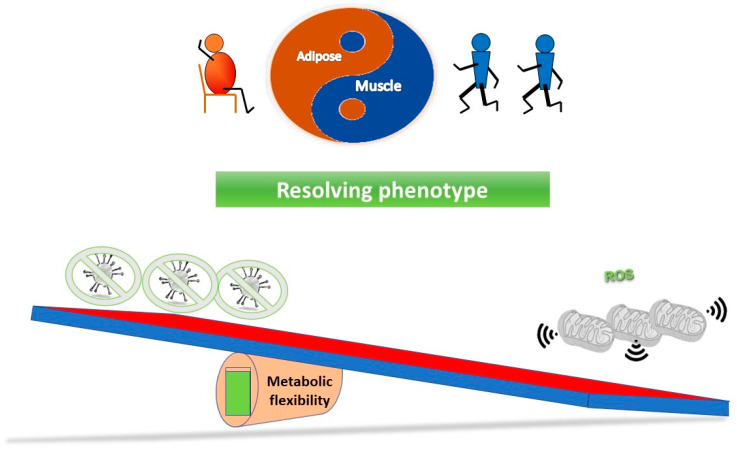
Why a good lifestyle may help against the long COVID. With mitochondria in optimal health, they have plenty of reserve capacity to ensure the metabolic flexibility to both ensure a good immune response, and also, for instance, via effective antioxidant capability, ensure inflammation is resolved.

Figure 3 outlines a simple concept in relation to a see saw tipping point; without sufficient starting metabolic flexibility, a host can get stuck in an inflammatory state. In contrast, Figure 4 indicates that with good mitochondrial health and metabolic flexibility, resistance to long COVID might be more likely.

### 3.1. The Sub-Optimal Mitochondrial Function Theory and Acute SARS-CoV-2 Severity

Several groups, including us, have been suggesting that prior sub-optimal mitochondrial function could be contributing to the morbidity when infected with SARS-CoV-2 as many viruses, including this one, can affect bioenergetics as it is a key part of their infectious modus operandi. The people thus most affected will likely be those who already have compromised mitochondria, for instance the elderly and those with underlying morbidities [22,23,24,25,104,105]. This could include a link to the reactivation of viruses like the Epstein–Barr Virus (EBV), which is well known to modulate mitochondrial function; we have suggested that this could result in a mitochondrial “double whammy” [106]. Indeed, data are beginning to support a link between long COVID and EBV reactivation [107]. There is also evidence that patients with primary mitochondrial defects (PMD) are also at greater risk, although they often have many other co-morbidities, including respiratory dysfunction [108]. The link between ageing and poor mitochondrial function has long been discussed, although the role of mitochondrial dynamics and hormesis indicate the relationship is more complex than thought [109].

### 3.2. Temporal-Compartmental Effects of the Virus vs. the Host on Metabolic Reprogramming

One problem with scientific acceptance of a significant role for mitochondrial function in COVID-19 is that its effects on metabolism are often varied in different tissues. However, new data are now providing some answers; it may well depend on when and where samples are taken from patients. For example, it appears that while mitochondrial bioenergetics is repressed in the nasopharynx, it is upregulated in the lungs. What seems to be happening is that the virus downregulates some aspects of oxidative phosphorylation, but in response, the host tries to upregulate them. In some tissues, such as the heart, the consequences of this repression can be severe because of the reliance on oxidative phosphorylation. Thus the timing during the infection is key—during active viral infection, it suppresses certain aspects of mitochondrial function to ensure production of metabolites for viral replication, for instance, upregulation of glycolysis and production of nucleotides, but as the virus is cleared, the system rebounds to repair [110].

This metabolic “Warburg” switch would be predicted to occur and does have similarities to what happens in cancer, and thus, why, potentially, anti-cancer and anti-viral strategies have some cross over as potential treatments [22]. A potentially interesting example of this may occur in the brain; data indicate that the virus can infect both astrocytes and neurons, but in astrocytes, it seems to enhance oxidative phosphorylation, depriving neurons of lactate and pyruvate. This seems to be tightly linked to the cognitive problems associated with this virus [13]. The reverse or inverse Warburg effect and metabolic coupling is critical in brain function, and its modulation could well be key in the development of Alzheimer’s [111].

Why this virus infects brain tissue is perhaps an interesting question. It could simply be a non-specific effect, but it could also be that by centrally modulating brain function, which can control inflammation, it may help in viral survival. For instance, by enhancing insulin resistance, so making more glucose and lipids available. Altering the behaviour of its host could also be an evolved strategy. It is certainly true that viruses have evolved to utilise their host’s machinery to make more viruses, which has gone hand in hand with modulation of mechanisms that control the immune system and metabolism to provide the materials, and energy that are needed. How they affect mitochondria depends on their replication rate, for instance, whether they induce, or prevent, apoptosis, and modulate metabolism. Some switch from one to the other to enable long-term infection, but most result in oxidative stress. It is likely that each virus has a different way of doing it [112].

However, if we accept that the host will reprogram its metabolism to try and get rid of the virus [43], which is likely linked to more general systemic effects related to the inflammatory-driven acute phase response (APR) and insulin resistance and alterations in lipid metabolism [113], then the boundary between “directly virus induced” vs. “host response to virus” becomes less clear. This definition becomes even more blurred with evidence that some proteins released during the APR can actually suppress inflammasome activation, hinting at the host trying to suppress excessive inflammation [114]. The metabolic link is perhaps further strengthened as COVID-19 not only worsens existing diabetes but can trigger it in people who did not previously have it; although some of the mechanisms mentioned above could be important, there is also some evidence of direct damage to the pancreas [115].

At the organ level this might mean that any tissue that has a high reliance on oxidative phosphorylation, which means in conventional terms that the Kreb’s cycle is running in the forward direction, could we well be at risk, especially if the virus can infect it, but also indirectly, if it is also susceptible to metabolic reprogramming during a generalised inflammatory response, or its supply of oxygen becomes compromised. Thus, the heart, neurons and the kidneys are likely to be very vulnerable. Certainly, renal problems associated with this virus are being increasingly recognised, especially, somewhat worryingly, in children [116].

In some respects, this could be viewed as a collateral non-specific metabolic reprogramming effect, for instance, of both the virus’s target cells and the immune system. At the cellular level, not only does this alter the death threshold of a cell, but it could well affect things like the generation of ATP to fuel autophagy. The potential problems of this latter point are perhaps highlighted by the mitochondrial-lysosomal theory of ageing. In effect, as mitochondrial function decreases and thus ATP production to drive the V-ATPase, the ability to remove damaged components, including sub-functional mitochondria by autophagy decreases—could lead to a vicious cycle. Post-mitotic long lived cells could well be particularly vulnerable, such as neurons, retinal pigment epithelium, cardiac myocytes and even skeletal muscle [117]. Proteomic studies of blood samples certainly indicating alterations in the autophagic pathway, which can, it seems predict long COVID [26]. In fact, it now seems that eye problems are becoming an increasing concern—even in the longer term [118]. Likewise, the link between skeletal muscle problems and COVID-19 are well recognised, and are associated with weakness and exercise intolerance, and could be related to direct and indirect effects, including motoneuron problems; the similarities to CFS are suggesting further investigational avenues. There is evidence of early mitochondrial dysfunction, akin to what happens to sepsis, and certainly, recovery from critical illness is strongly correlated with restoration of oxidative phosphorylation [119]. The root of this probably lies in the fact that mitochondria are central to the immune response [120].

The virus has also been associated with gastro-intestinal problems, which are partly related to acute severity, and in the longer term, disordered gut-brain interaction (DGBI) [121]. There are many similarities with COVID-19 infection and inflammatory bowel disease, in particular dysbiosis and its perpetuation after viral clearance—critically, it is thought that the gut could also be trophic for the virus as well. There are also similarities in metabolomic profiles, for instance in tryptophan as well as in some related to Kreb’s cycle compounds, including succinate [122]. The importance here is that it is now well recognised that there is bidirectional communication between the gut microbiota and mitochondria, for instance, via short chain fatty acids (SCFA), and other microbiota metabolites, including secondary bile products that can modulate mitochondrial energy and inflammatory function. In turn, mitochondrial function regulates gut function, inflammation, and gut wall integrity. Critically, the right amount of endurance exercise can beneficially modulate the gut microbiota, while excessive exercise can have the opposite effect [123].

Overall, this might suggest that much of the pathology could be related to a contextual metabolic reprogramming of different organ systems. Some could be related to a generalised immune response, but direct effects of the virus on some tissues would also be relevant, such as in the brain or the gut.

### 3.3. The Importance of Mitochondrial DNA Copy Number—A Tipping Point?

If we assume that one of the precipitating factors for long COVID is existing sub-optimal mitochondrial function, then one marker might be mitochondrial DNA copy number. One of the key themes emerging from this viral-attack/host-defence tug of war with regards metabolic reprogramming is that the host defence system has evolved, as a countermeasure, to induce destruction of some of its own components to help remove a pathogen. For example, programmed cell death (PCD) has evolved both as a mechanism to stop the spread of viruses, while, depending on the type of death, it can also act as a powerful inflammatory signal. This of course mirrors what happens in response to stress. The flipside is that viruses also manipulate PCD for their own purposes; mitochondria, because of the central role in many forms of PCD, are thus intimately evolved in this ancient arms race [124]. For instance, there are data suggesting that certainly in yeast, there is a programmed response that quite deliberately deletes mtDNA genes in response to oxidative stress in the short term to restrict production of ROS; this process could become maladaptive in the long term [30]. Certainly, it seems that mtDNA copy number (mtDNAcn) is important in overcoming heteroplasmic mtDNA mutation, which could be become a problem if not diluted out enough; in effect, the absolute levels of wild type mtDNA are key in determining pathology, especially in post-mitotic tissues, but not in rapidly dividing tissues [125].

Although there are some confounding data, the majority points towards mtDNAcn decreasing with age, which is probably associated with increasing mutation and heteroplasmy, and certainly, in contrast, healthy mitochondrial mass is associated with longevity [126]. This certainly fits with the mitochondrial bottleneck inheritance concept, in effect, a smaller number of heteroplasmic mitochondria when passed on during fertilisation will enable natural selection to take place between embryos, which may have relevance to the susceptibility and importance of mtDNA mutation in cancer [127]. When combined with the overall origins, and function of mitochondria throughout evolution, for instance, their role in longevity and apoptosis [128], their health could be pivotal in whether or not homeostasis is restored.

The implication is that for someone with poor mitochondrial health, the virus could easily precipitate an exaggerated inflammatory response, for instance, because their mitochondrial death threshold is lower, so more cells release mtDNA, which in turn, drives oxidative stress with further suppresses mtDNA gene expression, so tipping the system towards hypoxia and glycolysis. This would then lower mtDNAcn, and the effects of any detrimental mtDNA haplotypes could be become amplified.

Where this occurs is thus critical, for instance in organs with low replicative potential, such as in the nervous system, heart or brain, but it could also occur in the blood, in particular, in platelets (see below). This could result in a tipping point relating to a threshold. It could be argued that this could be determined, in part, by the prior mtDNA reserve. Evidence is that mtDNAcn change is associated with pathology, for instance, increased mtDNA copy number can be associated with better aerobic fitness (VO_2max_) and an active lifestyle and is greater than in someone living a sedentary lifestyle, with the lowest being seen in patients with T2D [32]. Of relevance is that negative effects of mitochondrial heteroplasmy because of mutations can be offset to some degree by increasing overall mtDNAcn [125]. Simply put, if a tissue contains low levels of mtDNA, some of which is mutated, then the likelihood of this mutation having a negative effect becomes more pronounced if there is a viral mitochondriopathy.

## 4. Mitochondrial Health and Platelets in Long COVID

In this section, we discuss, as it could be important in relation to clotting disorders, the potential role of mitochondrial health in relation to platelet function. Overall, the underlying concept being discussed in this paper is that of metabolically flexibility and reserve, and the ability of an organism to adapt to stress, which will be dependent on its mitochondrial health. This could include platelets.

In tissues such as the heart, brain or the immune system, poor mitochondrial health could clearly induce much of the pathology seen in these tissues. However, the platelet has also been identified as playing a role in the pathology of COVID-19. For instance, COVID-19 can directly induce platelet activation enhancing thrombosis [129]. Indeed, circulating mtDNAcn is being used to study mitochondrial health, with mitochondria coming from most circulating cells, including platelets. The pathology is associated with a “U” shaped numbers curve, which most likely reflects haematopoiesis. Although it has to be borne in mind mtDNA does not necessarily represent mitochondrial biology, as other factors can influence it [130]. Interestingly, genome-wide association studies (GWAS) of blood mtDNA have picked up associations with platelet activation, megakaryocyte proliferation and mtDNA metabolism [131], while platelet mtDNA methylation seems to be able to predict cardiovascular outcome in obesity [132].

Some of the most devastating pathologies associated with this virus, especially with hospitalisation, are associated with the increased risk of cardiovascular problems and venous thromboembolism (VTE) [133]. The risk of thrombosis is linked to platelet function, suggesting that if their mitochondria are unhealthy, then many of symptoms associated with both acute viral infection and long COVID, could be explained.

### 4.1. Platelets and Health

In humans, platelets are short lived anucleate cells that usually contain several mitochondria, which are essential for their haemostatic and immunological function. Critically, their activation can lead to apoptosis. Hence mitochondrial damage or dysfunction reduces their survival and can lead to an increased risk of thrombovascular events [19].

A key activating mechanism involves calcium modulation of the mitochondrial permeability transition pore (mPTP), which can lead to a loss of the mitochondrial membrane potential and exposure of negatively charged phosphatidyl serine (PS) at the platelet surface. Due to the heterogeneity of the platelet population, this process can be reversible in some, indicating that the overall response can be finely tuned [134]. They also, on activation, seem to activate aerobic glycolysis—but maintain mitochondrial function [135]. Suggestively, evidence indicates that the increased risk of coagulation in patients with Wiskott–Aldrich syndrome could be associated with smaller platelets and less mitochondria, and thus, less capacity to buffer calcium [136]. This is in keeping with the well described role of mitochondria in calcium homeostasis and signalling and certainly in platelets, fits with the data indicating the mitochondrial calcium uniporter (MCU) plays a role in regulating procoagulant platelet formation [137]. It also appears that dynamin-related protein-1 (Drp1), which is a GTPase involved in mitochondrial dynamics, is important in controlling platelet exocytosis [138]. A key point here is that as individual platelets only contain a small number of mitochondria, if any of them are damaged, or possibly, have mutations, then their health could dramatically affect platelet function. Data are showing that migrating cells can export damaged mitochondria via a newly described process called “mitocytosis” encapsulated in a in a “migrasome” [35]; whether platelets do this as well is an interesting question, but it seems likely (see below).

It is therefore suggestive that free mitochondria derived from platelets also modulate immune cells, suggesting that healthy mitochondria could have a translational treatment potential; this supports the emerging evidence that cells, in general, swap mitochondria as part of a homeostatic mechanism [139]. An interesting example of this is that activated platelets can transfer functional mitochondria to mesenchymal stem cells, enhancing their healing ability [140]. In contrast, mitochondria released from damaged brain tissue can induce platelet procoagulant activity [141]. It also seems that cancer chemotherapy can induce platelet dysfunction associated with mitochondrial damage [142]. Furthermore, cardiovascular disease is also linked to heightened platelet activation, but regular exercise reduces this effect [143]. In fact, it has now been shown that platelet mitochondrial function reflects systemic mitochondrial function following interventions such as physical activity training [144]. This reinforces the finding that mitochondria communicate with each other, for instance, via myokines, and play a key role in the systemic response to exercise [145].

In short, we should consider platelets as being part of the global mitochondrial system, which might suggest that their dysfunction could be important in say, disseminated intravascular coagulation (DIC), if, in general, a person’s mitochondrial function is sub-optimal. Equally, someone with an athletic disposition might well be protected, which does seem to be born out with the data [146]. In effect, it is possible that a healthy mitochondrial population engendered by exercise is likely reflected in platelets, while an unhealthy population as result of a poor lifestyle, is also reflected in these cells.

### 4.2. SARS-CoV-2, Platelets and Mitochondria

As indicated, a common finding in COVID-19 patients is hyperactivation of platelets. This could be due to increased inflammatory factors, but many viruses are also known to directly activate platelets and megakaryocytes, or indirectly via immune complexes. SARS-CoV-2 mRNA has been found in platelets, but there has been some discussion as to whether platelets express its main receptor, ACE2, although there are other platelet proteins it could bind to, such as CD147. Another route of uptake could be virus particles within extracellular vesicles (EVs) [147,148]. Indeed, new evidence seems to suggest that platelets from hospitalised patients can take up the virus via several routes, which does not always require ACE2. Intriguingly, this seems to result in PCD via apoptosis or necroptosis, but not pyroptosis; pyroptosis and necroptosis can be inflammatory, but apoptosis is normally not. Both viral proteins and fragmented RNA were detected, as well as ACE2 protein. Interestingly, it seems that the virus ended up in vacuoles, where it was digested, furthermore, there was no evidence of viral replication. It also seems that the virus induced platelets to release EVs. One explanation is that platelet uptake of this virus, and perhaps other viruses, is a way of neutralising these pathogens [149].

Given the well described role of mitochondria in several forms of cell death [150], this would indicate how mitochondrial health could determine outcome. Platelets have autophagic machinery, indicating they can remove dysfunctional components [151]; disruption of autophagy, for instance, due to poor mitochondrial function, could be pivotal in how they respond to stress. It is possible the virus could induce the platelet to release damaged mitochondria. Perhaps less appreciated, platelets, like most other cells, can also undergo apoptosis, which can also be modulated by mitochondrial health. They are also involved in multiple other functions, including immune surveillance, and are modulated by multiple compounds, including polyphenols [152].

If, as has been suggested, platelets are acting as a means of neutralising the virus, this raises the interesting possibility that this has been selected for during evolution as these cells do not have nuclei. Many viruses modulate nuclear traffic, and as 99% of mitochondrial protein is coded for in the nucleus [153]; this could be important. Thus, this evolutionary strategy perhaps reduces the ability of the virus to reprogram this organelle. Certainly, it seems that like many viruses, SARS-CoV-2 can metabolically reprogramme many cells towards glycolysis, in effect, a kind of Warburg shift (aerobic glycolysis) that enhances viral replication—which may explain why diabetes can predispose to worse disease [92,154]. This suggests that a Warburg shift in COVID-19 could be critical [22]. It may therefore be relevant, as indicated, that platelet activation results in a switch to aerobic glycolysis and an upregulation of the pentose phosphate pathway (PPP) and increased ROS generation via NADPH oxidase (NOX), where mitochondrial function remains viable but inhibition of respiration does not inhibit activation; suppression of aerobic glycolysis is thus a developing approach to preventing excessive platelet activation and thrombus formation [135]. This of course represents changes in how the Kreb’s cycle is functioning.

In terms of a possible mitochondrial swapping between cells, data suggest that SARS-CoV-2 can infect brain endothelial cells where internalised spike protein components can directly damage mitochondria [155]. In turn, extracellular mitochondria released from damaged brains can induce platelet activation via binding to phospholipid-CD36 [141]. Furthermore, in COVID-19 patients, peripheral blood mononuclear cells (PBMC) are reprogrammed, displaying compromised mitochondrial function [156]. In contrast, platelet mitochondria can reprogramme CD4+ T cells, which could be important in the suppression of the proliferation of PBMCs [139]. Overall, this seems to suggest there is an inflammatory/anti-inflammatory feedback system involving mitochondria, platelets, and infected cells.

Perhaps a further clue may come from the observation that mitochondria contain components of the renin-angiotensin system (RAS), which includes ACE2—the antioxidant and anti-inflammatory component of the RAS that produces Ang (1–7). In neurons, this is involved in generating nitric oxide (NO), and the whole system, including ACE2 and a newly identified receptor, the mitochondrial Mas-related receptor (MrgE), seems to decrease with age [157]. Interestingly, ACE2, delivered via exosomes, can restore endothelial function by restoring mitochondrial health following exposure to angiotensin II [158]. It is thus perhaps noteworthy that as serious COVID-19 disease is thrombotic it can be viewed through the prism of Virchow’s triad of vascular damage, altered blood flow and hypercoagulability, where the RAS plays a central role. Of relevance is the fact that ACE2 is a key negative regulator of this system, including in platelets via production of NO [159].

Overall, this might suggest that if platelets are not functioning properly due to sub-optimal mitochondria, this could be part of a positive feedback loop driving continual inflammation in someone with long COVID. If, as is becoming clear, mitochondria are being swapped between cells and tissues, then this could reflect a systemwide sub-optimal mitochondrial population, which is expressed in platelets as well.

### 4.3. The Optimal Platelet Mitochondrion—Exercise as a Medicine

A key question of course is if the platelets in people with long COVID do, in part, reflect a persistent sub-optimal systemic mitochondrial induced pathology, how might it be reversed? One universal treatment that seems to help in multiple indications is physical activity.

Acute exercise can activate platelets, but regular exercise diminishes this response, and favourably modifies their function at rest—and likely plays a key role in prevention against cardiovascular disease [143]. Critically, platelet mitochondrial function seems to reflect their overall systemic status [144]. In short, exercise results in a healthier platelet mitochondrial population. However, the truth is that exercise is a key component of maintaining mitochondrial health throughout the body, not just in skeletal muscle [160].

A healthy mitochondrial system results in good control of their potential to elicit an immune response. Due to their bacterial ancestry, release of both mitochondrial RNA, DNA, and proteins can amplify an immune response; this often occurs during oxidative stress [161]. A key modulator of this is mitophagy [117]. This system is tightly integrated with the immune system, for instance, via the production of interferons to defend against viruses [162], of which MAVS are central following activation of pathogen pattern recognition receptors [163]. MAVs also interact with the NOD-like receptor pyridine containing (NLRP) 3 inflammasome, which also detects pathogenic nucleotides, and can generate ROS and IL-1β [164]. Mitochondrial function is thus key in viral defence, in particular, in relation to interferon signalling.

Of relevance in relation to a poor lifestyle possibly leading to worse acute disease and long COVID, is that sterile activation of NLRP3 may occur in the metabolic syndrome, leading to generalised inflammation (“metainflammation”), which is also associated with mitochondrial dysfunction [165]. Platelets contain NLRP3, so it is interesting that its activation by the Dengue virus might result in thrombocytopenia [166], as well as in primary immune thrombocytopenia; tellingly, this is also associated with reduced anti-oxidant capacity [167]. As there is an association between the metabolic syndrome and severity of COVID-19 [168], this might suggest that a heightened activity of NLRP3 caused by a pre-existing condition might further worsen platelet function when infected by the virus.

Conversely, not only does exercise protect against the metabolic syndrome, but aerobic fitness seems to provide a measure of protection against developing severe COVID-19—which is likely related to mitochondrial fitness [169,170]. Exercise stimulates adaptive improvement of mitochondrial function in muscle, and via myokines, in other tissues throughout the body [145]; acute exercise is inflammatory, but following recovery, enhances an overall anti-inflammatory phenotype [171]. The underlying principle is hormesis; stimulating mitochondrial function can be associated with oxidative stress, which in turn, induces an adaptive increase in both mitochondrial and anti-oxidant capacity—which seems to be essential for a balanced inflammatory response [172]. Data seem to suggest that macrophage mitochondrial function is key during injury, for instance, mitochondrial ROS is pro-inflammatory to begin with, but via hormesis, results in an anti-inflammatory milieu enabling resolution [173].

This would seem to suggest that to some extent, sub-optimal mitochondrial function in platelets may well reflect that of the entire body and potentially be associated with clotting imbalances. Critically, it would also hint that exercise could well play a key role in both prevention and treatment of conditions like long COVID.

## 5. Discussion and Implications

Given the evolutionary roots of eukaryotic cells, it is no surprise that mitochondria are central to every aspect of modern cell functioning and have thus been part of a billion year plus arms race with viruses. Most of the symptoms of long COVID can be explained, to varying degrees, by sub-optimal mitochondrial function in multiple organs, including, we suggest, in platelets. In some respects, the metabolic/inflammatory phenotype is not too dissimilar to that induced by a poor lifestyle, which might explain why this population is more susceptible [22]. The potential scale of this interaction is perhaps indicated by data that suggest in the USA, between 2017 and 2018, only 6.8% of adults displayed optimal cardiometabolic health [174]. What is a normal metabolic system and what is an optimally healthy one may not be the same thing in this modern world.

It has been said that as an ancient oxidative symbiont, if not constantly stimulated, the mitochondrion slowly loses its ability to handle oxidative and macronutrient stress, leading to the palette of lifestyle induced disorders we see today, which hints at why endurance exercise is so important [175]. Hormesis is in fact a vital component of maintaining health, as it is this very process that has driven evolution and adaptation, and without it, large sections of society appear to be experiencing accelerated ageing phenotype [176]. It could therefore be argued that long COVID, at the broader level, is simply what happens when this new virus meets a metabolically unprepared host.

**Figure 5:**
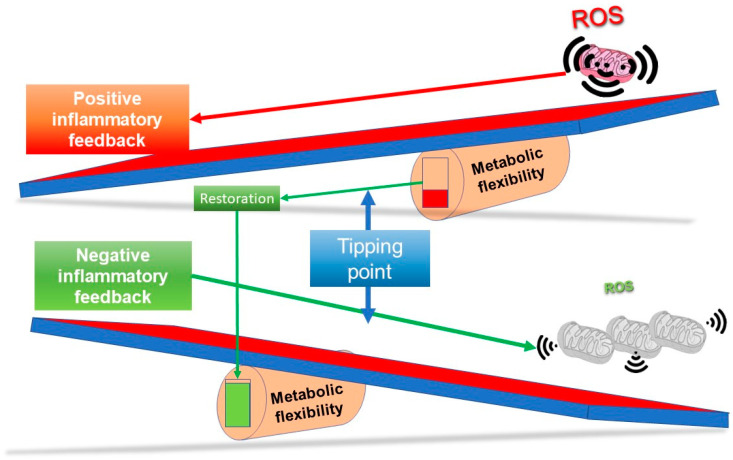
Tipping the system back to health. As long COVID might be represented by the upper see saw whereby the metabolic flexibility fulcrum is too far to the right to enable the restoration of homeostasis, the system has effectively passed a tipping point and got stuck. Resolution may revolve around enhancing metabolic flexibility and/or suppressing inflammatory pathways to shift the fulcrum to the left, as in the lower see saw. At the simplest level, someone starting with a reduced population of slightly stressed mitochondria could well be more likely to develop long COVID—which implies, for resolution, that the system needs to be induced to adapt towards normal by hormetic approaches known to stimulate renewal of a healthy population of mitochondria. The most obvious of these is a carefully balanced prescription of physical activity.

In this last section, we look at long-term health, a possible mitochondrial definition of long COVID, and how we might approach treatment. The simple observation is that just about all strategies that have been used to improve functional longevity, and thus, a healthy lifespan and potentially slow the ageing process, may well help in long COVID. It is quite possible that any mild stressor, if applied in the right way, could be beneficial. This of course not only includes exercise, but also calorie restriction, as well as diet, but also things like acute temperature stress—both hot and cold. Indeed, one of very first insights into hormesis came from heat stress and the role of heat shock proteins [177], which is perhaps supported by data on sauna use, coupled with cold stress (e.g., rolling in snow), and longevity [178]. Figure 5, which combines components from Figure 3 and Figure 4, illustrates one way of viewing how improving mitochondrial health and thus metabolic flexibility, can resolve inflammation restoring system homeostasis.

### 5.1. Can We Say What Long COVID Is?

At its simplest, it could be described as a chronic and self-perpetuating metabolically imbalanced dyshomeostatic state induced by the virus that fails to resolve. It perhaps has all the hallmarks of an accelerated ageing syndrome and thus similarities to inflammaging. It could be argued that this occurs because the original system was not robust enough, and because the virus might induce a particular set of metabolic conditions associated with inflammation, it can tip a weakened system into a positive feedback spiral—which might be made worse because part of the host’s defence system against the virus also acts in a similar metabolic direction. The key here is that the capacity of a single system, represented by the Kreb’s cycle and thus, mitochondria, must have enough reserve to operate in multiple modes in different tissues. In a few words, it requires metabolic flexibility.

Inflammation is triggered in several ways, but oxidative stress, and thus, ROS, are pivotal, and initially work, beyond a threshold, as a feed forward amplification loop. It could be said that resolution occurs because the very same stress activates a feedback suppressive anti-inflammatory/antioxidant system that engenders repairing damage once the threat is removed—the right amount of exercise is a good example of how this can be induced [179]. One chronic driver of inflammation could be that damaged mitochondria are releasing bits of oxidised DNA, which is thought to be an inflammatory mechanism as it can activate the NLPR3 inflammasome [180]. Again, this could come back to a vicious cycle outlined in the mitochondrial/lysosomal theory of ageing [117]. Of course, where the line is drawn between chronic inflammation, and say, adaptive immunity is perhaps less clear. A key component of immunity is its ability to remember past challenges, for instance, via epigenetics, but this can become maladaptive [181]. Does this virus somehow alter the epigenetic memory?

One possibility is that the human homeostatic system evolved over many generations where its metabolically flexible state was reliant on stimulation from a hormetic environment, for instance, lots of physical activity, occasional fasting, temperature extremes and a diet high in bioactive plant compounds. In effect, the system became “canalised” to a particular environment. In a modern world, for large sections of the population because of technology, this stimulus has been lost, resulting in reduced antioxidant capacity and tendency for the immune system to over-react in response to infections. Without the induced metabolic flexibility, the memory signal becomes much stronger and chronic, so potentially becoming maladaptive. As organisms age, this restorative pathway naturally declines and robustness decreases, and they become increasingly frail and less able to adapt. Thus, the concept of “inflammaging” emerged, which also explains how immunosenescence develops; mitochondrial function and hormesis are pivotal in modulating this [182].

This would certainly suggest an inability to restore redox balance, which has similarities to what happens in CFS [183], could play an age-related role in developing long COVID. Certainly, evidence is that nutrient sensing, mitochondrial function, stem cell exhaustion and altered cellular communication are all linked to the epigenetic aging process, although interestingly, it appears that cellular senescence, telomere shortening and genomic instability may not be [184]. However, there is some evidence that shorter telomeres are linked to worse COVID-19 severity in relation to T cell expansion [185]. It also seems that the DNA damage response (DDR) increases with age-related telomere shortening, which seems to increase ACE2 expression [186]. In contrast, genetically predicted short telomere lengths have also been shown not to be related to severity [187]. This may indicate that telomere length alone, especially if genetically determined, is not enough to explain it—indicating there is a strong age and environmental element.

Clearly, loss of immune system function is an important factor in ageing, and “immunosenescence” is a well described ageing problem, as it results in a reduced ability to resist pathogens and cancer cells and critically, can drive senescence in other organs [188]. Loss of mitochondrial function is part of the process in immunosenescence, but it is likely that mild mitochondrial stress, via the production of mitokines, does have an anti-inflammatory function and can limit immunosenescence overall [189]—which fits well with the importance of hormesis in keeping an optimally functional immune system. Indeed, it is becoming clear that skeletal muscle plays a vital role in maintaining a healthy immune system [190]. For instance, it can harbour anti-viral CD8^+^ T cells, so preventing T cell exhaustion [191].

Ultimately, it could argued that as a result of an “intelligence paradox”, whereby humans have all but removed all the stressors required to induce an adaptable and flexible metabolism [192], they have shifted their overall metabolic phenotype to one that favours the virus, and even if they manage to clear the virus, the system cannot always reset properly.

### 5.2. Restoring Metabolic Flexibility—A Hormetic Approach?

The implication of hormesis is that our systems do require stimulus from the occasional right kind of mild stress to be optimally healthy. If long COVID is fundamentally a chronic and self-perpetuating metabolically imbalanced dyshomeostatic state/syndrome induced by the virus that fails to resolve, can the right stimulus, or inhibition of feed-forward inflammatory pathways help in recovery? This could take the form of drug-based therapy, exercise, calorie restriction, or even, electromagnetic and light-based modulation of metabolism. The goal would be to restore mitochondrial health.

In fact, because of the lack of consensus on the underlying causal pathology and a plethora of definitions, there are already many trials underway trying different approaches targeting both individual symptoms, such as fatigue or lung problems, or more generalised approaches, which, because of the inflammatory component are centred around suppressing inflammation. For instance, approaches include hyperbaric oxygen and radiofrequency-based therapies, corticosteroids and statins, anti-inflammatory biologics, dietary supplements ranging from vitamin C, nicotinamide to coenzyme Q10 and melatonin, polyphenols and targeting Nrf2, to exercise and breathing classes [193,194]. Interestingly, a Kreb’s cycle intermediate, oxaloacetate, has also been tried, with some success in both ME/CFS and long COVID associated fatigue [195]. Even an endocannabinoid due to its anti-inflammatory potential have been studied—with some evidence of efficacy [196].

In this section, we review some of the main approaches being tried, many of which are known to enhance functional longevity and health. Some of these are likely to be reliant to some degree on hormetic mechanisms, while the mechanism of others relies more on direct modulation of wayward pathways.

#### 5.2.1. A Healthy Plant-Based Diet?

It has been suggested that a healthy plant-based diet could be important in treating long COVID [197]. Plant rich diets contain lots of phenolic compounds, which are well known to modulate mitochondrial function in a number of ways, including being “hormetic” [198]. Why many plant compounds act as medicines is perhaps a much deeper question, but a clue may be that they may have started out as sunscreens, in effect, dissipating solar potential, which meant during evolution they became integrated into a generalised stress response. This would explain why they modulate so many intracellular targets, including mitochondria, and have clear dose-related differential effects, as well as anti-pathogen actions, and the ability to control host cell fate. It also explains why they can be both direct antioxidants and oxidants [198]. In effect, they can buffer a potentially highly damaging redox situation, and turn it into an adaptive signal, and tip the organism out of a vicious cycle.

#### 5.2.2. Antioxidants and Oxidants—The Metformin Experience

Aside from anti-viral drugs, the concept that biology relies on hormetic stressors to maintain optimal health may provide some insight to the success, or not, of some treatments. For instance, many “antioxidant” drugs have been given to restore redox balance, but they have had limited success in COVID-19 [183]. One reason could be that direct antioxidants simply inhibit the hormetic response. Modulation of redox, however, is still a potential approach, especially if targeted to the right intracellular location to manage the balance between adaptive signalling and suppression of excessive, and potentially damaging, oxidative stress. This is of course could be one way of viewing how plant-derived phenolic compounds might work. However, there are some more traditional drugs that may work in a similar way, for example, metformin.

Metformin has several intracellular targets, a primary one is complex 1 of the ETC with some reports suggesting it can inhibit ROS production at this complex [199]. However, it has also been shown to be mitohormetic via enhancement of ROS, leading to increased longevity [200]. It has been investigated in COVID-19 and has shown some benefit in hospitalised patients [201,202,203]. A possible mechanism has been suggested involving inhibition of mitochondrial function and inflammasomes, which could help to reduce pulmonary inflammation [204]. Perhaps significantly, it has also been shown that metformin can reduce platelet hyper-activity in patients with polycystic ovary syndrome, which also appears to involve stabilising mitochondrial function [205]. Metformin is therefore an example of a mitochondrially targeted compound with several effects; the outcome is likely to depend on dose and the metabolic/inflammatory status of the cell and its mitochondria.

This is pivotal, as it provides an insight into how to suppress oxidative stress-driven inflammation, while also potentially, stimulating ROS-based adaptation. Thus, the finding that RET can be induced by TNF, which generates mtROS at complex 1 from succinate, is perhaps significant, as the effect can be blocked by metformin [56]. Given that different compartments in the body could be experiencing different grades of inflammation and resolution and have variable metabolic flexibility due to where the virus has infected, it is possible to explain how one compound could, in theory, both inhibit inflammation in one place, while stimulating mitochondrial regeneration in another compartment. If mitochondria and their components are indeed undergoing a constant “do-si-do” amongst cells, then as long as one compartment can start to regenerate healthy mitochondria, then this could be key in regenerating metabolic flexibility and resolution throughout the entire body.

#### 5.2.3. Learnings from Physical Activity

The global mitochondrial health concept does suggest the enhancing mitochondrial health and capacity in one organ could result in enhancement in other parts of the body. One of the problems of a drug-based approach is that even if many of these compounds do act pleiotropically, for instance, modulating inflammation, they are unlikely to be able to induce a global adaptive response. In contrast, it is now becoming recognised that one of the most generic and powerful medicines is exercise, especially as overall it can enhance anti-inflammatory mechanisms [169,170,206,207,208].

However, like all medicines, it needs to be prescribed carefully, as getting the “dose” right could be critical. The uncertainties over its use is perhaps highlighted as NICE has advised against graded exercise for patients recovering from COVID-19 [209]. However, many clinical practitioners have suggested its use should be based on risk stratification [210]. The clue here is that exercise is hormetic [211], and thus follows a “U” shaped curve—if the system is very heavily compromised, it will be unable to adapt quickly, and too much will clearly induce inflammation without adaptation and make it worse.

It could therefore be argued that COVID related exercise intolerance is just an extreme example of a bad case of delayed onset muscle soreness (DOMS). In many respects, the approach is thus no different to standard athletic training practices, whereby untrained individuals need to build up capacity by carefully managing exercise bout intensity with sufficient rest periods to enable adaptation, so, with time, enhancing their ability to resist the oxidative stress induced by increasing amounts of heavy exercise. This would certainly suggest that at least initially, high intensity interval training (HIIT) is not a good idea, but low-level endurance training, keeping within the aerobic threshold, is optimal, especially after a period of rest. Determining when to do this is maybe key, so may require measuring both more standard biochemical parameters to determine physiological status, as well as performance metrics, such as VO_2_ max, balance, grip strength and walking speed.

Related to this is perhaps a line of research that investigates those biochemical pathways involved in the exercise response to identify targets for new “exercise mimetics” [212]. The concept of “exercise in a pill” has been around for a while, in particular, for compounds that modulate the AMPK-SIRT1-PGC1α pathway, for instance, metformin, epcatechin, resveratrol and AICAR [213]. Again, however, the dose of this compound is probably key in its actions and how it modulates mitochondrial function as they are biphasic [214].

#### 5.2.4. Calorie Restriction and the Ketogenic Diet—Sirtuins to the Rescue?

Another approach is to restrict calories, as this is well-known to reverse many metabolic dysfunctions. For instance, a ketogenic diet stimulates mitochondrial function [215]. Indeed, calorie restriction, which is not only a way to enhance lifespan, but also has a whole slew of other beneficial effects, including stimulating autophagy and mitochondrial renewal, has been suggested as a possible way to help in COVID-19 [216].

Calorie restriction and autophagy are pivotal for ageing research. Many calorie restriction mimetics show similar metabolic effects, some of which, like rapamycin, directly modulate a key pathway involving mTOR that is key in controlling autophagy in response to calories [217]. One of the pivotal pathways underlying the benefits of calorie restriction involves Sirt1, the sirtuins and NAD. This has led to the investigation of using NAD^+^ precursors as calorie restriction mimetics, which can have anti-inflammatory effects, such as nicotinamide mononucleotide (NMN) in various indications, especially in ageing individuals as NAD^+^ production decreases with age [218]. Another NAD^+^ precursor is nicotinamide riboside (NR), which has also been shown to have many benefits, and has been investigated for SARS-CoV-2 [219]. Sirtuins are NAD-dependent deacetylases that act as metabolic sensors, and are key in modulating mitochondrial function and antioxidant systems in response to nutrient stress, for instance, Sirt1 activates PGC1α, which is a key regulator of energy metabolism and master regulator of mitochondrial biogenesis [220].

What is perhaps less appreciated is that sirtuins are also highly conserved anti-viral factors [221]. One group in the anti-viral response are members of the poly (ADP-ribose) polymerases (PARP), that use NAD as a source of ADP-ribose (ADPR) which they covalently link to target proteins. They are upregulated by interferon. Critically, it seems that SARS-CoV-2 genome encodes for an ADPR hydrolase, hence reversing the output of the PARPs. This therefore dramatically upsets the cell’s NAD metabolome, driving down levels of NAD^+^ and NADP^+^, which would explain why compounds like NMN and NR could have antiviral activity [222]. It has been suggested that COVID-19-induced sepsis could be associated with severe mitochondrial dysfunction, in which suppression of sirtuin activity and enhancement of HIF-α activity could play a key role [223]. This would certainly fit with this imbalance, if the person survived, being a causative factor in long COVID.

#### 5.2.5. Emerging Nonchemical Modalities

There are also emerging new treatments, such as photobiomodulation (PBM), which uses low intensity longer wavelengths of light to modulate metabolism via absorption at various complexes in the ETC of mitochondria, such as cytochrome C oxidase, or possibly, in ion channels. Hormesis seems to be one mode of action, and it is now being used for several conditions, but a commonality is as an anti-inflammatory intervention [224]. However, the dose, timing and wavelength are going to be important, for example, light at 980 nm shows some very interesting dose effects and seems to work via complexes III and IV, inhibiting ATP production at low power, but stimulating at higher power (0.1 vs. 0.8 watts, respectively), inversely, it stimulated superoxide production at the lower power to a much greater extent than the high power [225]. Its use in wound healing is also becoming accepted, including treating orals lesions in patients with COVID-19 [226].

Then there is the use of static magnetic and electric fields, which when applied in the right way, seem to be able to suppress inflammation—possibly via a hormetic mechanism based on altering quantum spin-states. For example, this technique has been used to control aberrant redox signalling and inflammation in a mouse model of T2D [227]. Indeed, because of the emergence of new technologies, the concept of “quantum biology” and the observation that life is both using, and is sensitive to, electromagnetic fields is rapidly gaining acceptance [228]. Because of their ability to have anti-inflammatory and tissue healing effects, which seem to be partly reliant on modulating mitochondrial function, it could well have application in long COVID as well.

#### 5.2.6. Mitochondrial Transplant

However, what if a person’s “healthy” mitochondrial population is so depleted that they simply cannot tip back into health—even in their muscles? Is it possible that a person could simply lose enough healthy mtDNA that the system may never recover? Mitochondrial transplantation has been tried for several disease associated with mitochondrial dysfunction, and is certainly an area of interest, but there are many challenges to overcome, not least induction of an inflammatory response, the source and preservation, and the delivery method. Although autologous transplantation is the preferred option, allogenic mitochondria would be more readily available, but potentially more problematic [229]. As far as the authors are aware, it has not been tried for diseases like COVID-19.

#### 5.2.7. Can We Measure Mitochondrial Health?

A key underlying aspect of this paper is that sub-optimal mitochondrial health prior to infection may, to some degree, determine the likelihood of developing long COVID and potentially, also be a biomarker of recovery and guide treatment. Showing that there is a correlation with the clinical phenotype, especially as someone recovers, could be very informative. For example, comparing mitochondrial function with markers of inflammation, oxidative stress and symptoms, and physical functionality, may well help in determining the dose and timing of treatment. The problem is not only defining what mitochondrial health is, but also accurately measuring it to produce a distribution curve across a population in, ideally, a way that is non-invasive, or at the very least, from a blood sample. For example, determining the ATP/ROS ratio in say, peripheral blood mononuclear cells (PBMCs).

Would studying Kreb’s cycle intermediates and other blood metabolites help to track mitochondrial function in long COVID? The data outlined in this paper do suggest that there are clear changes in the metabolic profile. One interesting observation is the comorbidity-associated glutamine deficiency that predisposes to severe COVID-19 as it compromises many immunological functions [230]. The main source of glutamine is active muscle, although it can be produced by other tissues such as the liver—and it has been long known that physical activity is key to proper immune function and stress resistance. It is also vital for fast growing cells as it provides carbon for the Kreb’s cycle, as well as being a precursor for glutathione production and thus protection of mitochondrial function [231]. However, the Kreb’s cycle is also a source of glutamine, although it can be produced by branched chain amino metabolism, plus, its production can also be manipulated (limited) as an anti-pathogen strategy, as well as to suppress inflammation, in turn, pathogens also manipulate its metabolism for their own ends [232]. In short, mitochondrial function plays a key role in glutamine metabolism.

Studies have compared permeabilised skeletal muscle cell mitochondrial function with circulating blood cells, such as PBMCs and platelets. The underlying premise being that mitochondrial respiration in circulating cells can indicate a person’s overall metabolic health. Although in one study it seemed that circulating cells could not replace muscle biopsies, it did suggest that some parameters of platelet cell mitochondria did reflect muscle cell mitochondria, such as complex 1 leak and oxidative phosphorylation coupling efficiency—so could be of some value [233].

In fact, is has been suggested that the link between cardiometabolic risk factors and altered immune cell function is via reduced respiratory capacity, and a recent study did show this in apparently healthy individuals with some risk markers—hinting at immune cell reprogramming towards glycolysis [234]. Platelets can also be used to measure mitochondrial function, are easier to isolate than PBMCs, and they do seem to reflect overall systemic mitochondrial function; initial studies in runners before and after an event do seem to support this [144,235]. So mitochondrial health in platelets could well be something that could be measured and relevant.

Totally non-invasive ways of measuring mitochondrial function are still not available. However, autofluorescence and lifetime imaging, say, of NADH and FAD^+^, which can not only give good images, but also tell us a great deal about bioenergetics of live cells is an emergent technology that is being rapidly developed [236]. Having a handheld device that could measure bioenergetics of blood flow in capillaries under exposed skin may eventually provide us with a possible way forward.

## 6. Conclusions

Long COVID is likely, for many people, to be the result of a viral challenge to an already less robust system that is itself the outcome of the removal of environmental factors that are required to maintain mitochondrial health through stress adaptation, or potentially, have been compromised by a co-morbidity. There will also be gene–environment interaction—everybody’s immune system is different, not just genetically, but also because of the challenges they have faced from injuries, infections, and other trauma during their lifetime.

As a generalisation, it could well be that many people can be “tipped” into a chronic dysmetabolic/inflammatory cycle. The similarities to an accelerated ageing phenotype suggest that approaches used to slow the ageing process, and enhance functional longevity and a healthy lifespan, can be applied by understanding the underlying pathways and the concept of hormesis. However, because the system only has so much capacity to recover, which decreases with age, treatments need to be measured and commensurate with the person’s current mitochondrial health status and ability to adapt. They could range from calorie restriction to exercise and natural products, and some drugs, but also new emerging therapies such as photobiomodulation. In some cases, direct inhibition of some pathways may be required to break the system out of a vicious cycle, for instance, by suppressing inflammatory pathways, or potentially, supporting antioxidant systems.

## Data Availability

Not applicable.
